# Antiviral and antibacterial peptides: Mechanisms of action

**DOI:** 10.1016/j.heliyon.2024.e40121

**Published:** 2024-11-05

**Authors:** Mahdyeh Neghabi Hajigha, Bahareh Hajikhani, Maryam Vaezjalali, Hossein Samadi Kafil, Raana Kazemzadeh Anari, Mehdi Goudarzi

**Affiliations:** a Student Research Committee, School of Medicine, Shahid Beheshti University of Medical Sciences, Tehran, Iran; b Department of Microbiology, School of Medicine, Shahid Beheshti University of Medical Sciences, Tehran, Iran; c Drug Applied Research Center, School of Medicine, Tabriz University of Medical Sciences, Tabriz, Iran

**Keywords:** Antimicrobial peptide, Drug resistance, Antibacterial peptide, Antiviral peptide, Mechanism of action

## Abstract

Antimicrobial peptides (AMPs) present promising alternatives for addressing bacterial and viral multidrug resistance due to their distinctive properties. Understanding the mechanisms of these compounds is essential for achieving this objective. Therefore, this comprehensive review aims to highlight primary natural sources of AMPs and elucidate various aspects of the modes of action of antiviral and antibacterial peptides (ABPs). It emphasizes that antiviral peptides (AVPs) can disrupt the replication cycle of both enveloped and non-enveloped viruses at several stages, including pre-fusion, fusion, and post-entry into the host cell. Additionally, the review discusses the inhibitory effects of ABPs on bacterial growth, outlining their extracellular actions as well as their intracellular activities following membrane translocation. Factors such as structure, size, electric charge, environmental factors, degrading enzymes, and microbial resistance against AMPs can affect the function of AMPs.

## 1 Introduction

The growing prevalence of multidrug-resistant (MDR) microorganisms has heightened the risk of epidemics caused by these agents, making it a pressing public health concern worldwide. In response to this crisis, various strategies have been recommended, including the identification and development of new antimicrobial compounds [1]. A report released by the World Health Organization (WHO) in 2019 outlined a list of multidrug-resistant bacteria, highlighting the urgent need to create new antibacterial agents to combat them [2]. Drug resistance is not confined to antibacterial agents; health organizations have also raised alarms about resistance to antiviral drugs, particularly among immunocompromised patients. Furthermore, existing antiviral medications do not provide comprehensive coverage against all viruses and often prove insufficiently effective [3]. In this context, antimicrobial peptides (AMPs) have emerged as promising new agents against MDRs, with their applications expanding rapidly.

AMPs are characterized by structures consisting of 8–15 amino acids and are recognized as one of the earliest mechanisms of innate immunity across many life forms [4]. Beyond their roles in innate immunity, AMPs also function as modulators of the adaptive immune system in higher eukaryotes [5]. These small proteins are predominantly positively charged, owing to their high content of lysine and arginine, which enables them to interact with negatively charged surfaces and bacterial membranes [6]. Their broad spectrum of activity and specific functional properties have led to the consideration of AMPs as viable treatment options for persistent infections. In recognition of the significance of these compounds, the Antimicrobial Peptide Database (https://aps.unmc.edu/) has been established to catalog and introduce AMPs based on their sources and functions. As of now, this database has documented 3324 AMPs.

Nevertheless, the application of these materials is restricted due to challenges related to stability, cost, toxicity, bioavailability, specificity, and half-life [7]. Research is currently underway to prepare these peptides for clinical use, seeking to enhance their effectiveness through structural modifications or combinations with other compounds. In conjunction with other studies, it is crucial to investigate the specific mechanisms of action of each peptide. This study aims to provide a brief overview of select antimicrobial peptides (AMPs) and to precisely elucidate the mechanisms of action of antiviral peptides (AVPs) and antibacterial peptides (ABPs).

## 2 Source of AMPs

**Fig. 1:**
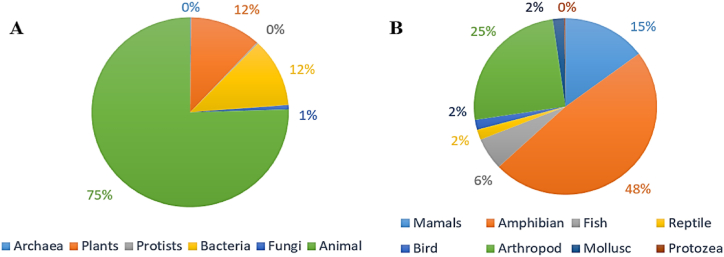
Statics of the antimicrobial peptides. (**A**) Source of antimicrobial peptides (in total: 3569) and (**B**) animal derived antimicrobial peptide percentages (in total: 2600) in the antimicrobial peptide database https://aps.unmc.edu/in December 2023.

Almost all organisms utilize antimicrobial peptides (AMPs) to defend against pathogens and competitors (Fig. 1A). A significant proportion of the 2600 peptides cataloged in the aforementioned database are derived from animal sources, with approximately 1117 types produced exclusively by frogs. Additionally, 146 AMPs associated with humans are synthesized by immune and tissue cells (Fig. 1B). AMPs are typically extracted and purified from their sources using chromatography-based techniques such as high-performance liquid chromatography (HPLC) [8], thin-layer chromatography with direct bioautography (TLC-DB) [9], and liquid chromatography-mass spectrometry (LC-MS) [10] among others. This section summarizes some of the most notable natural sources of peptides, including plants, animals, and bacteria, that are of interest for drug discovery.

Plant AMPs, characterized by a molecular weight of around 10 kDa (kD) and containing 4 to 12 cysteine residues, are classified into three families: thionins, defensins, and cyclotides [11]. These peptides are found in the stalk, roots, seeds, flowers, and leaves of plants, offering protection against environmental microorganisms [12]. AMP-rich crude extracts have exhibited antibacterial activity (e.g., Pteleopsis suberosa against *Helicobacter pylori*), antifungal activity (e.g., Santolina chamaecyparissus against *Candida* spp.), and antiparasitic effects (e.g., Cowpea against *Leishmania amazonesis*) [13]. Therefore, the manipulation and incorporation of transgenic plants capable of AMP production hold significant potential in medicine and plant pathology.

Most invertebrates, particularly insects and marine invertebrates, contain AMPs in their hemolymph, hemocytes, phagocytes, and epithelial cells [14,15]. Several of these peptides, such as cecropins [16], tachyplesin [17], and drosocinin [18], have been identified to possess antibacterial (against both Gram-positive and Gram-negative bacteria), antifungal, and antiviral properties, functioning as components of the innate immune system.

In vertebrates (such as fish, reptiles, birds, and mammals), AMPs are produced by phagocytic white blood cells as well as epithelial cells of the skin and mucous membranes. They prevent microbial invasion through two mechanisms: by being stored in the granules of phagocytic granulocytes and mast cells, or by existing freely in mucosal and skin secretions and bodily fluids [19,20]. In contrast to other organisms, AMPs in vertebrates also play roles in modulating and regulating acquired immune responses, cancer progression, and male reproduction, in addition to their functions within the innate immune system.

The major mammalian antimicrobial peptides (AMPs), which include those produced by humans, belongs to cathelicidins, defensins, and AMPs produced by platelets and the liver [21].

Cathelicidins possess a conserved cathelin-like N-terminal domain and are stored in an inactive form within the granules of leukocytes [22]. Upon stimulation of reservoir cells, these peptides are processed and released, exhibiting bactericidal properties [23]. While there are various mammalian AMPs within this family, LL-37 is the sole cathelicidin identified in humans [24]. LL-37 features an alpha-helical structure that contributes both antibacterial and regulatory functions [25].

Defensins are antimicrobial and chemotactic peptides divided into three subtypes—alpha, beta, and theta—based on the arrangement of their disulfide bonds [26]. These disulfide bonds enhance the stability of peptide structures, thereby reducing susceptibility to proteolytic degradation [27]. Additionally, defensins can activate the complement system via the classical pathway [28]. Only alpha and beta defensins are present in the human body. Beta-defensins are produced by a wide range of cells, including leukocytes, epithelial cells, and cardiac and muscle cells, whereas alpha-defensins are exclusively synthesized by neutrophils, macrophages, and intestinal Paneth cells [29].

**The third group**: of mammalian AMPs is produced by platelets and the liver, which are less extensively studied compared to the other two types. Examples of human AMPs in this category include thrombocidins TC-1 (a platelet AMP) and Hepcidin 20 (produced in the liver) [21].

AMPs in bacteria, referred to as bacteriocins, enhance microbial survival in competitive environments [30]. Based on their source of production, bacteriocins are generally divided into two main groups: those produced by Gram-positive bacteria, which can be further categorized into lentibiotic and non-lentibiotic AMPs, and those produced by Gram-negative bacteria [31].

Despite the diverse sources of AMPs, most encounter challenges in clinical application due to issues such as instability, high costs, limited bioavailability, short half-lives, toxicity, and lack of specificity [7]. In recent years, various strategies have been employed to improve their effectiveness and specialized use while minimizing limitations. Characterizing the structure and physicochemical properties of AMPs can facilitate the discovery of new peptides. Additionally, approaches ranging from modifying existing AMPs to designing new peptides, as well as utilizing delivery systems for AMPs, have been explored.

## 3 Antiviral activity

Peptides exhibiting specific virucidal activity have emerged as promising candidates in the design of antiviral drugs. Antiviral peptides (AVPs) are derived from a diverse array of natural sources and can also be identified through biological methods, such as high-throughput screening and computational approaches, all of which are cataloged in the Antiviral Peptide Database (AVPdp) [32,33].

Enveloped and non-enveloped viruses pose distinct characteristics, that affect the level of resistance to antiviral drugs. Enveloped Viruses have a host cell derived lipid membrane (envelope) that contains viral proteins essential for entry into host cells [34]. Non-Enveloped Viruses lack an envelope and consist only capsid (a protein coat) that encases their genetic material. This structure exhibits greater robustness compared to enveloped viruses [34].

Enveloped Viruses typically enter host cells via fusion with the host cell membrane, a process that can be disrupted by drugs targeting the envelope [35]. However, non-Enveloped Viruses often enter cells through endocytosis [35]. Therefore, they are less susceptible to membrane disrupting drugs that target lipid membranes but more vulnerable to ant-capsid agents.

Accordingly, resistance to Antiviral Drugs is different between enveloped and non-enveloped viruses. Enveloped viruses exhibit resistance through fusion inhibitor or lipid-disrupting tolerance and higher mutation rates [36]. As well as, the robust nature of the capsid and limited target options may lead to challenges in developing effective antiviral treatments against non-enveloped viruses [37].

**Fig. 2:**
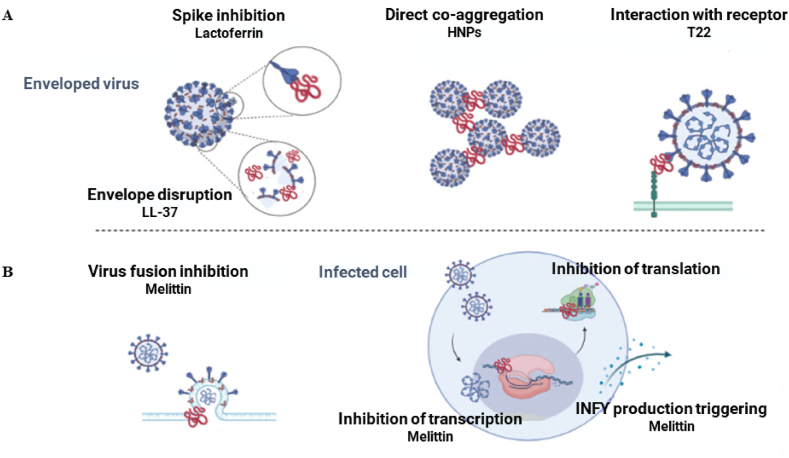
Potential mechanisms of antiviral peptides (AVPs) against enveloped viruses. Part **A** includes pre-fusion antiviral activities of cationic peptides that prevent viral-cellular interaction, and **B** represents fusion and intracellular antiviral activity of AVPs.

Taking together, due to more membrane-destroying activity and anti-fusion properties of AVPs, the highest antiviral activity has been reported against enveloped viruses; particularly against the Human Immunodeficiency Virus (HIV), Hepatitis B Virus (HBV), Hepatitis C Virus (HCV), and Influenza A Virus (IAV) [38]. The pre-fusion, fusion, and intracellular activities of AVPs are briefly illustrated in Fig. 2A and B.

### 3.1 Anti-enveloped virus activity

The antiviral effects of AVPs can be categorized into two primary mechanisms: extracellular and intracellular. AVPs can bind to viruses and neutralize them due to their specific features, such as electric charge or shape, which allow them to match external attachment sites on the virus.

Lactoferrin, a natural peptide from the transferrin family secreted in various body fluids, exhibits an anti-Human Cytomegalovirus (HCMV) effect attributed to its positive charge [39,40]. This peptide demonstrated a strong anti-HIV effect when negatively charged groups were added to it [41], This enhancement is likely due to the ability of the charged-modified protein to bind to the gp120 V3 loop [42], which increases the net negative electric charge of the virus and inhibits virus-cell binding and fusion. Additionally, lactoferrin's anti-HCV function is linked to its direct interaction with the E1 and E2 proteins of the viral envelope [43]. Studies have shown that viral particles are neutralized before they can absorb and enter hepatocytes [44].

Furthermore, LL-37 binds to and aggregates viruses, such as herpes simplex virus type 1 (HSV-1) and respiratory syncytial virus (RSV), leading to pore formation and destruction of the viral lipid bilayer membrane [45,46].

Another antiviral mechanism observed in certain defensins is viral aggregation. Human neutrophil peptides (HNPs) can inhibit viral infectivity by promoting the aggregation of viral particles [47]. Research indicates that treating viruses with HNPs before inoculation into epithelial cells significantly reduces their attachment rate compared to treatment administered after inoculation [48]. Another class of defensins, known as retrocyclins, appears to exert an even stronger antiviral effect than HNPs, utilizing a similar mechanism to induce aggregation of the influenza virus [49].

Binding to cellular surface receptors is a critical first stage in viral infection and plays a determining role in disease inhibition. Consequently, many peptide-based antiviral treatments focus on blocking or disrupting these receptors. For instance, in the context of COVID-19, which is caused by the SARS-CoV-2 virus, the interaction between the spike protein and human angiotensin-converting enzyme 2 (hACE2) is essential for viral entry into cells [50]. Researchers have been working on designing and synthesizing peptides that can disrupt or inhibit these receptors to prevent infection onset. One potential treatment involves creating peptide-based inhibitors that compete with the virus spike by mimicking all or part of its receptor [51]. Biomimetic peptides containing the virus-binding domain of ACE2 have shown significant anti-SARS-CoV-2 activity in vitro [52]. T22, a synthetic peptide related to the promiscuous peptide tachyplesin, has demonstrated inhibitory activity against HIV-1 and HIV-2 in vitro. It is suggested that T22 specifically binds to the chemokine receptor CXCR4, which is necessary for HIV entry into CD4^+^ T cells, thereby blocking effective attachment [53,54].

Following receptor binding, enveloped viruses continue the infection process by fusing their viral envelope with the cell membrane, facilitating the release of viral contents into the cell cytoplasm. Matanic et al. observed that melittin, a peptide isolated from insect venom, blocks viral entry by interfering with this fusion step in HSV-1 [55]. Melittin is believed to inhibit cell fusion by interacting disruptively with the Na+, K + ATPase, a cellular enzyme that plays a role in membrane fusion [56]. Conversely, Temporin G, an amphibian AMP, impairs the function of the influenza hemagglutinin by neutralizing the virus's cell entry [57]. This peptide blocks the conformational rearrangement of the HA2 subunit, which is essential for the fusion of the viral envelope with the intracellular vesicle membrane, without reducing HA expression levels [57].

Once inside the cell, the virus can replicate its nucleic acid using both viral and cellular enzymes and components. This replication step can be directly inhibited by destructive or blocking peptides, such as inhibitors targeting protease, polymerase, integrase, helicase, and other factors involved in transcription and replication. For example, Kerstin et al. identified a peptide derived from influenza A, with a single influenza B-specific amino acid substitution, that demonstrated inhibitory efficacy against the influenza virus polymerase complex. This polymerase complex consists of PB1, PB2, and PA subunits, and the dual-binding peptide binds to the PA subunit, thereby disrupting the protein-protein interaction between PB1 and PA [58]. Additionally, some AVPs can indirectly disrupt the normal cycle of viral transcription by altering intracellular signaling pathways related to viral replication. For instance, melittin, as highlighted earlier, may significantly decrease HIV-1 transcripts by activating phospholipase A2 [59] or, alternatively, by reducing the activities of calmodulin [60] and protein kinase C [61]. This peptide might disrupt HIV regulation by manipulating the activity of intracellular transcriptional stimulatory factors, such as NF-kB, AP-1, and NFAT, or promoting interferon-related inhibitory pathways [62].

### 3.2 Anti-non enveloped virus activity

While most AVPs primarily target enveloped viruses, there is increasing evidence that cationic peptides can effectively combat non-enveloped viruses as well. Antiviral cationic peptides (12–50 amino acids) are positively charged and amphipathic agents, which contain cationic residues, such as lysine and arginine [63]. So they can disrupt or aggregate the viral particles by targeting the viral negatively charged envelope, capsid, and other components. They also can bind to viral proteins or cell surface receptors, inhibiting their function and preventing the virus from attaching to host cells or entering them [64]. Moreover, they may cause Inhibition of viral replication and modulation of host immune response, by directly interfering with viral or host cell nucleic acids, enzymes, and other intracellular proteins with negative charge [64]. Most AVPs are cationic, though some exhibit anionic or non-cationic properties [65].

Non-cationic peptides are neutral or negatively charged, which probably have less tendency to bind with virus particles. Their possible antiviral action may be more related to interfering with viral replication cycles and modulating the immune system. Several examples of effective AVPs derived from various sources have been reported against non-enveloped viruses with different actions. The cecropine B and synthetic form CF17 showed the disintegration of the viral capsid [66]. They inhibited infectious pancreatic necrosis virus (IPNV) known as a fish pathogen. Similarity, CAP37 demonstrated antiviral activity against adeno virus by capsid rupturing [67]. Aggregation and capturing the virions instead of disrupting the capsid are function of HD5 (Human alpha defensin) and RTD-1 to prevent BK and Papilloma virus from binding and entering the cells [68,69].

The peptide Epinecidin-1 (Epi-1) has been shown to inactivate the foot-and-mouth disease virus (FMDV), which belongs to the Picornaviridae family, by interfering with its uptake into target cells and inhibiting viral transcription following cell entry [70]. Additionally, Di Biase et al. demonstrated in an in vitro assay that a peptide derived from bovine lactoferrin (bLfcin) can prevent adenovirus binding to HEp-2 cells by occupying the viral binding site located on the cell surface [71].Inhibition of endosomal acidification and blocking virus replication is another mechanism that has been used by CAVPs P9 and P9R against human rhinovirus infection [72]. Another notable AVP is LVLQTM, a 6-amino acid peptide that exhibits ex vivo and in vivo inhibitory effects against human rhinoviruses (HRV) and human enterovirus 71 (EV71) by blocking the viral 2A cysteine protease (2Apro) [73,74]. This peptide acts as a pseudosubstrate, interacting with the substrate-binding pocket of the enzyme, which is critical for viral replication.

## 4 Antibacterial activity

Bacteria interact with cationic peptides primarily through electrostatic interactions. This is attributed to the distinct anionic components found in both Gram-negative and Gram-positive bacteria, such as lipopolysaccharides (LPS) in Gram-negative bacteria and teichoic acids in Gram-positive bacteria, as well as the plasma membrane in both types [75]. Consequently, antimicrobial peptides (ABPs) can exhibit multifunctional or broad-spectrum activity, making them attractive candidates for the development of antibacterial agents [76,77].

In addition to broad-spectrum activity, peptides that target a specific effect site are also crucial as selective antibiotics against particular pathogenic agents [78]. Historically, the antibacterial potential of peptides was thought to be limited to mechanisms such as membrane penetration or perforation leading to the release of cytoplasmic contents into the extracellular environment [79]. However, recent research has revealed various extracellular functions for these compounds, and it is now recognized that some peptides can enter cells without disrupting the membrane, representing a novel mode of action [21].

### 4.1 Mechanism of antibacterial action of AMPs

**Fig. 3:**
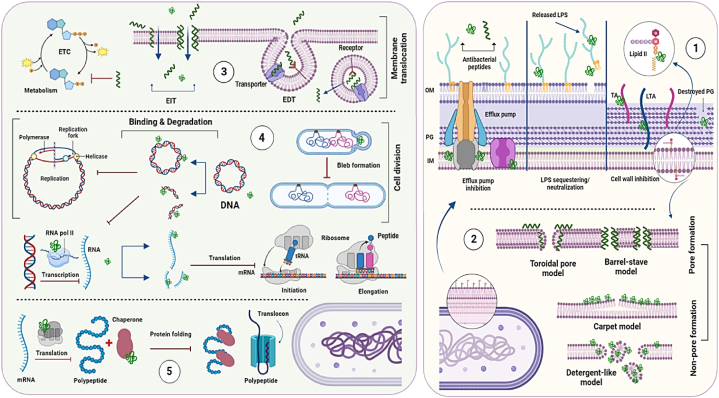
The schematic scheme of antibacterial action of AMPs. Extracellular antibacterial activity of AMPs is shown in right, which represents non-membrane (**1**) and membrane (**2**) action model. Left side shows Intracellular antibacterial activity of AMPs after translocation through the membrane. Based on interaction site they can inhibit bacterial energy metabolism system (**3**), cell division, replication, and transcription (**4**), translation and protein folding (**5**). OM: outer membrane; PG; peptidoglycan; IM; inner membrane; ETC: electron transport chain; EIT: energy independent translocation; EDT: energy dependent translocation.

AMPs generally exert bactericidal effects directly interacting with bacterial membranes or other components. However, these peptides indirectly inhibit pathogenic bacteria by regulating the immune system. Fig. 3 provides a visual representation of the antibacterial effects of cationic peptides. Figs. 3–1 and 3-2 indicate non-membrane and membrane action model respectively. Figs. 3– shows intracellular antibacterial activity of AMPs after translocation through the membrane.

#### 4.1.1 Extracellular activity (cell surface)

##### 4.1.1.1 Non-membrane activity of AMPS

###### 4.1.1.1.1 Anti-biofilm antimicrobial peptides

**Fig. 4:**
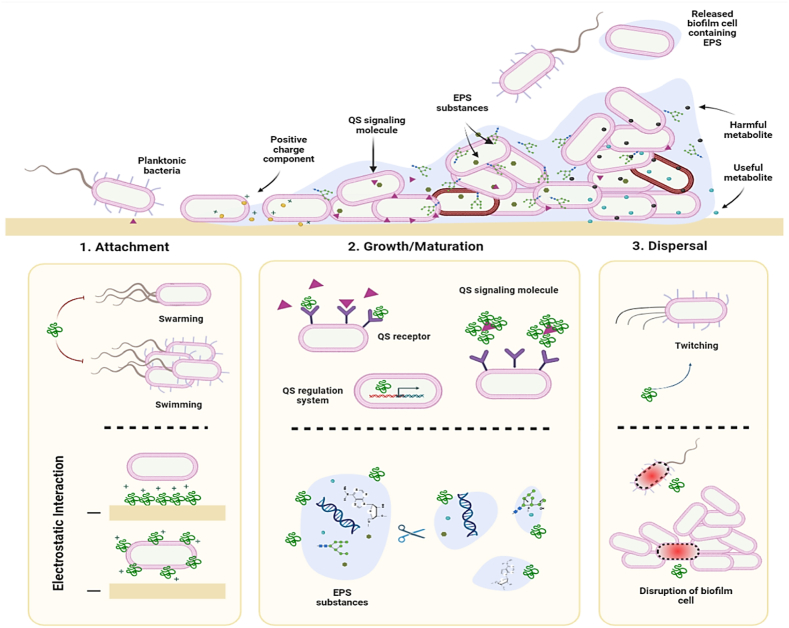
Possible anti-biofilm effect of AMPs in different stages of biofilm development process. As shown in the above, biofilm formation consists of three main stages. Peptides may interface with enhancer and inhibitor factors to block this process or degrade biofilm three-dimensional structure. Furthermore, in some conditions, such as electrostatic interactions, the promoting effect is not unexpected.

Biofilms are extracellular polymeric substance (EPS) that consist predominantly of polysaccharides, proteins, and various other compounds excreted by bacterial communities, which protect bacteria and inhibit the access of antimicrobial agents to deeper layers [80]. Biofilm-encased bacteria exhibit 10–1000 times greater antibiotic resistance compared to planktonic forms, establishing biofilms as a primary factor in persistent bacterial infections [81]. Therefore, peptides that can deactivate these components should not be overlooked. Various peptides that interfere with different stages of biofilm formation have been described, although their precise mechanisms of action remain largely unknown (Fig. 4).

Among the many factors involved in biofilm formation, quorum sensing has emerged as a crucial regulator in some bacterial species, attracting increasing attention in the study of inhibitory peptides. For instance, the LL-37 peptide has been shown to cause *Pseudomonas aeruginosa* to downregulate genes essential for biofilm development by interfering with the Las and Rhl quorum sensing systems [82]. Similarly, BCp 12, a peptide derived from milk, demonstrates antibiofilm activity through its interaction with the peptide receptors of the Agr system in *Staphylococcus aureus* [83].

Bacterial motility during the early stages of biofilm formation can facilitate attachment to surfaces, while at later stages, it may contribute to the instability of the biofilm structure [84]. Research has demonstrated that LL-37 and cathelicidin-related AMP (CRAMP) disrupt biofilm establishment and stability by modulating swarming motility at different developmental stages [82,85]. Their effects may arise from changes in charge; for example, these peptides may increase biofilm formation by creating a positive charge that enhances binding to negatively charged bacteria, an area that requires further investigation [86]. Additionally, other peptides have been developed to prevent biofilm formation by blocking quorum sensing and interfering with protein trafficking [87]. Furthermore, peptides can degrade existing biofilms, facilitating their elimination by interacting with extracellular polymeric substances [87].

Nevertheless, biofilms demonstrate a variety of resistance mechanisms that help them to endure the effects of antimicrobial peptides (AMPs) [88]. The EPS matrix acts as a physical barrier that limits the penetration of AMPs into the biofilm. This matrix can trap AMPs, reducing their effective concentration at the microbial cell surface. Bacterial cell within biofilm often exhibited reduced metabolic activity and a dormant state, which can provide less susceptibility to AMPs. Genetic Adaptation, quorum Sensing system, active extruding efflux pumps, nutrient limitation and stress responses, and environmental factors are the other strategies involved in resistance of biofilms.

###### 4.1.1.1.2 Anti-capsule antimicrobial peptides

The presence of capsules in bacteria, such as *Klebsiella*, poses a challenge by limiting the access of antimicrobial peptides (AMPs), including immune system peptides and polymyxins, to the bacterial surface. This is akin to the role of the extracellular matrix in biofilms. In this context, Renee et al. designed a peptide known as PepW, which effectively induced capsule aggregation and destruction [89]. Interestingly, despite the observed changes in capsular structure due to binding, there was no significant decrease in capsular attachment [89].

###### 4.1.1.1.3 Efflux pump inhibitor peptides (EPIPs)

Efflux pumps are critical factors contributing to antibiotic resistance, as they are located in the membrane and cell wall of bacteria and can expel antibiotics from within the cell. While these peptides may not directly inhibit bacterial growth, they can serve as adjuvants to prevent the expulsion of antibiotics or to inhibit biofilm formation [84]. A considerable number of peptides exhibiting pump inhibitory effects have been reported [90].

For instance, a cyclic cationic antibiotic-sensitive peptide (CASP) was found to interact with the periplasmic region of MtrD in *Neisseria gonorrhoeae*; however, it did not sensitize the bacterial cell to antibiotics [91]. This peptide demonstrated resistance to efflux, unlike previously established cyclic peptides such as LL-37 and colistin [92,93]. In a related study, Michel et al. synthesized a peptide-based pump inhibitor that interacts with the TM4-TM4 binding region of the Pseudomonas SMR pump, effectively disrupting its mediated efflux [94]. Similarly, peptides designed to mimic the transmembrane domains TM1 or TM8 of AcrB were used in *E. coli*, resulting in destabilization by targeting membrane-embedded interactions [95].

**AMPs Acting on LPS, Lipoproteins, Lipoteichoic Acids, and Peptidoglycan** The differences in cell wall composition among bacterial groups significantly influence the modes of action of antimicrobial peptides (AMPs). In Gram-negative bacteria, the outer membrane (OM) serves as the outermost layer of the cell envelope, providing protection from the external environment. The OM consists of a unique arrangement of lipids, with phospholipids in the inner leaflet and lipopolysaccharides (LPS) in the outer leaflet. In contrast, Gram-positive bacteria lack an outer membrane and possess a thicker peptidoglycan layer relative to their Gram-negative counterparts. Additionally, Gram-positive bacteria contain lipoteichoic acids (LTA)—a class of amphiphiles that play a crucial role in their structural integrity.

Both Gram-negative and Gram-positive bacteria also include other significant amphiphilic compounds, such as lipoproteins (LP), which have recently been recognized for their importance. While these compounds are not essential for bacterial survival, they greatly influence immune system stimulation when released or when they bind to cell receptors during infection.

Amphiphilic properties of cell wall components facilitate the binding affinity of AMPs to bacterial membranes. The hydrophobic regions of AMPs interact with the lipid components, while the hydrophilic regions can interact with the polar head groups of LPS or LTA [96]. Given this context, along with anti-cell wall and membrane peptides, it is essential to consider single-target or dual-purpose antimicrobial peptides that specifically inhibit these compounds [97]. Such advancements could lead to significant progress in infection control and prevention of sepsis and septic shock. In this section, we will discuss peptides targeting the bacterial cell wall and their interactions with amphiphilic components.

###### 4.1.1.1.4 Interactions of AMPs with lipopolysaccharides (LPS)

LPS is a critical component of the cell wall in Gram-negative bacteria, forming a complex with the lipopolysaccharide-binding protein (LBP) and CD14 receptor to interact with TLR4 on macrophages [98]. This binding triggers inflammatory pathways and contributes the onset of systemic inflammatory response syndrome (SIRS) [99]. Immune system peptides, such as cathelicidin, lactoferrin, NK-lysin, and Limulus anti-LPS factor (LALF), are capable of binding to and disrupting LPS aggregates; thus, they are classified as antiendotoxin and anti-inflammatory peptides [[100], [101], [102]].

Currently, there is a growing interest in synthesizing multifunctional peptides that possess characteristics such as antimicrobial activity, LPS/lipoprotein sequestering or neutralizing effects, and immunomodulatory properties. This study aims to develop multifunctional peptides that simultaneously exhibit antimicrobial activity, LPS sequestering/neutralizing capabilities, and immunomodulation.

Previous researches have indicated that acylation of lactoferricin-derived peptides, such as LF11, enhances their antitoxin and antimicrobial activities [[103], [104], [105]]. Although some acylated derivatives like P2-15, P2-27, P2-29, and P2-33 did not display antimicrobial properties, they effectively reduced and eliminated biofilms of *Pseudomonas aeruginosa* [105]. This effect may be attributed to the high affinity of alginate, a main component of the *Pseudomonas* biofilm, for hydrophobic compounds [106]. Additionally, the increased molecular weight of acylated peptides may hinder their passage through the extracellular matrix of the biofilm, limiting access to the bacterial wall.

Notably, *Brucella abortus*, a Gram-negative bacterium with an atypical LPS and cell wall structure, exhibited greater sensitivity to acylated peptides compared to their non-acylated counterparts [107]. In vivo studies have shown that the interaction between acylated compounds and LPS reduces lethality in animal models [103,108], indicating that these compounds are more effective at neutralizing LPS compared to non-acylated peptides. In terms of LALF, alterations in polypeptide chain length [109] or conformation from linear to circular structures [110] can affect its antibacterial and anti-endotoxin properties. For example, a cyclic form with disulfide bridges demonstrated enhanced cytokine inhibitory activity compared to linear peptides [111]. Another study noted that a slightly shorter peptide exhibited lower anti-endotoxic activity but increased antibacterial efficacy [112].

Classic membrane-active AMPs, such as polymyxin B and melittin, also have LPS-sequestering and neutralizing effects. Other structurally unrelated lipopeptides, like gramicidin S, tyrocidin, efrapeptin, and DOPER, maintain anti-LPS properties. Additionally, studies have shown that polyamines can bind to lipoteichoic acid (LTA), inhibiting TLR2-mediated NF-κB activation.

Based on in vitro and in vivo models, the antibacterial activity and LPS and LTA neutralization potency of these peptides correlate with the length of the acyl chain. Polyamines with longer acyl chains tend to exhibit diminished inhibitory effects on LPS and Gram-negative bacteria compared to Gram-positive bacteria. In drug design, acylpolyamines are highly regarded due to their multitarget nature, which may lead to slower development of resistance compared to other antimicrobial agents. Furthermore, these polyamines are likely to be safer for clinical use due to their propensity to selectively bind to specific bacterial structures, such as LPS and LTA.

###### 4.1.1.1.5 Peptidoglycan-targeting antimicrobial peptides (AMPs)

Peptidoglycan is a mesh-like polymer that encases the bacterial membrane, providing protection and maintaining cell shape [112]. Peptides that impact peptidoglycan are categorized into two main groups: (a) Peptidoglycan Synthesis Inhibitor AMPs: These AMPs bind to essential structures involved in cell wall formation. A key target is lipid II, a conserved precursor molecule critical in the biosynthesis of peptidoglycan. Defensins (such as Plastacin and HNP-1), bacitracin, and vancomycin are known to specifically target lipid II [113]. Defensins interact with lipid II via its negatively charged pyrophosphate moiety [114]. Since lipid II is primarily important for cell wall structure in Gram-positive bacteria, some of these peptides may exhibit limited activity against Gram-negative bacteria [115]; (b) Peptidoglycan-Degrading Peptides: These peptides act to degrade the existing peptidoglycan structure. For instance, research by Michaela et al. introduced RWRWRW-NH2, a synthetic hexapeptide that compromises cell wall integrity by inhibiting the respiratory chain and delocalizing MurG, a key protein involved in lipid II biosynthesis [116].

###### 4.1.1.1.6 Other inhibitory peptides

A recently published study described a synthesized peptide that inhibits the invasion of *Listeria monocytogenes.* This peptide may block the internalization of *L. monocytogenes* by interfering with the interaction between internalin A and E-cadherin, as suggested by molecular docking studies [117].

##### 4.1.1.2 Membrane activity of AMPs

Despite the diverse range of cationic antimicrobial peptides (AMPs) and their various sites of action, most exhibit a strong affinity for negatively charged cell membranes. Consequently, many FDA-approved AMPs, including gramicidin, daptomycin, oritavancin, telavancin, and colistin, are classified as membrane-active [118]. Their interactions with potential electrical gradients can instigate membrane disruption and facilitate cell entry [119].

However, the precise mechanisms of action (MOAs) of these membrane-active peptides remain unclear, hindering efforts to fine-tune their selectivity for specific bacterial strains. Research on electrostatic interactions and molecular dynamics has given rise to models that elucidate and guide the design of specific mechanisms of action. Luo et al. and colleagues have proposed two primary models based on the formation of holes in the peptide-induced membrane arrangements [120]: (a) Transmembrane Pore Model: This model comprises two subtypes—the barrel-stave model and the toroidal pore model. Both arise from the insertion of oligomers into the lipid bilayer [121]. When monomeric peptide molecules reach a particular threshold concentration, they aggregate on the membrane's surface, interacting with hydrophobic headgroups to create a transmembrane channel. This channel facilitates osmotic lysis by allowing the passage of water and ions. Notable examples of natural pore-forming peptides include gramicidin, colistin, melittin, maculatin, and alamethicin [118].

(b) Non-Membrane Pore Model: This model encompasses the Carpet model and the detergent-like model. In contrast to the transmembrane pore model, peptides in this model interact with phospholipids as monomers, aligning parallel to the membrane's surface and effectively carpeting it. At high concentrations, the rotation of these peptides disrupts the phospholipid balance, leading to membrane rupture and the formation of micelles [122]. Examples of peptides that function in a detergent-like manner include daptomycin, colistin, LL-37, aurein 1.2, and piscidin 1 [118].

#### 4.1.2 Intracellular activity

Antimicrobial peptides (AMPs) can enter bacterial cells without causing damage to their membranes, potentially disrupting various intracellular processes. These AMPs, often referred to as cell-penetrating peptides (CPPs), can enter cells through both energy-dependent and energy-independent mechanisms [87].

**Energy-Independent Entry Mechanisms**: Two primary methods of energy-independent direct permeation include: **Formation of Instantaneous Pores**: Certain peptides, such as Buforin 2, can create pores in the membrane, allowing for direct entry [123], and **Direct Translocation via Membrane Instability**: Peptides like Cateslytin can translocate through membranes that become unstable [124,125].

##### 4.1.2.1 Energy-dependent entry mechanism

In energy-dependent pathways, endocytosis is mediated by membrane protein receptors, facilitating the transport of peptides into the bacterial cell. For example, PR-39 and Bac7 are known to enter cells via SbmA, a recognized transporter [126].

As well as, macropinocytosis is a non-clathrin and non-caveolar energy-dependent mechanism for non-specific cellular uptake of CPPs [127]. Common examples include Tat peptide (derived from HIV-1) [128], P3I7 and P3L7 (designed chimeric peptides) [129], that can trigger macropinocytosis.

Upon entry, these peptides can interfere with various cell cycle processes, leading to alterations in cell division. For instance, exposure of *Escherichia coli* to HD5ox, a human defensin, induces bleb formation at the cell pole or division site [130]. Additionally, certain AMPs can inhibit transcription and replication through RNA polymerase inhibition. Examples include Microcin J25 and capistruin [131], which block transcription processes.

Other peptides, such as indolesine and TO17 (a TFPI-1 C-terminal peptide), inhibit and disrupt hereditary substances—namely RNA and DNA—ultimately impeding nucleic acid biosynthesis and causing the formation of filamentous *E. coli* [132,133]. Some proline-rich AMPs are particularly potent, capable of completely halting protein synthesis by interfering with translation and ribosome-associated processes [134]. Specific peptides target different phases of protein synthesis: **Initiation**: Peptides like Onc112, pyrrhocoricin, and metalnikowin inhibit the transfer of aminoacyl-tRNA (aa-tRNA) to the ribosomal A site. **Termination**: Apidaecin 1b and Api 137 obstruct the 70S ribosome, preventing the release of factors during termination [135]. Moreover, synthesized proteins require chaperone-associated folding, typically mediated by the DnaK/Hsp70 system in bacteria [136]. This has led to DnaK becoming an attractive target for designing peptide-based antibacterial agents. Researchers have recently developed proteomimetics that bind to DnaK in a multi-domain targeting approach, creating DnaK-selective and species-specific peptides [137]. Additionally, specific peptides, such as Bac7, possess distinct targets that influence various stages of protein synthesis [135,138].

Antimicrobial peptides (AMPs) play a crucial role in regulating the intestinal microbiota and maintaining the integrity of the intestinal epithelium, where intestinal stem cells (ISCs) are pivotal for tissue homeostasis and regeneration [139]. AMPs modulate the local microenvironment, promoting ISC maintenance and function [140,141].

Additionally, AMPs can influence the proliferation and differentiation of ISCs into various intestinal cell types, such as enterocytes, goblet cells, and paneth cells, depending on the specific AMP and the surrounding conditions [142]. They modulate several key signaling pathways known to control ISC proliferation and differentiation: Wnt signaling pathway induces ISC proliferation and self‐renewal, BMP signaling pathway regulates ISC differentiation, and Notch signaling pathway maintains ISCs and balances secretory and absorptive progenitors [143].

For instances α-Defensins (e.g., HD5) and RegIII (Regenerating Islet-Derived Protein) are produced by Paneth cells, known as stem cell guardians, in the intestine and have been shown to play a role in the maintenance and proliferation of ISCs [142]. Other example is Lactoferrin, which enhances the proliferation and differentiation of ISCs into mature intestinal cells by activating Wnt signaling [15].

## 5 Factors affecting the action mechanism of antiviral and antibacterial peptides

Several structural and environmental factors can influence the mechanism of action of antibacterial and AVPs. Besides, this article aims to assist researchers by elucidating the mechanisms of action of peptides, thereby enhancing their clinical application through structural modifications and other effective strategies. Consequently, a comprehensive summary of these factors is described below.

### 5.1 Structural characteristics

The primary structure of peptides, including their amino acid composition and sequence, significantly impacts their biological activity. Antiviral and antibacterial peptides often possess a high proportion of hydrophobic and cationic residues. This unique composition facilitates interactions with microbial membranes, leading to disruption and cell lysis. For instance, the presence of specific motifs can enhance binding affinity to target cells, influencing the peptide's efficacy [144].

### 5.2 Peptide length

The length of peptides plays a critical role in their mechanism of action. Generally, shorter peptides may penetrate microbial membranes more effectively [145,146], while longer peptides might exhibit enhanced specificity and stability [147]. The balance between these two aspects is crucial; thus, optimizing peptide length can improve therapeutic outcomes.

### 5.3 Charge and hydrophobicity

The net charge of peptides affects their interaction with negatively charged microbial membranes. Cationic peptides, for example, can bind to the membrane, leading to pore formation and subsequent cell death. Hydrophobicity also contributes to membrane disruption; as hydrophobic regions of the peptide interact with lipid bilayers [148,149]. Recent studies indicate that AMPs with high hydrophobicity and amphipathic characteristics correlate with hemolytic activity [150]. Therefore, the selection range of these peptides must be optimized to improve antimicrobial effectiveness and minimize host cell toxicity.

### 5.4 Environmental factors

The biological environment, including pH, temperature, and metal ions, can influence peptide stability and activity [151]. For example, certain peptides may exhibit optimal activity at specific pH levels, affecting their ability to interact with target cells [152]. Additionally, variations in temperature can alter peptide conformation, impacting their functional efficacy [153].

### 5.5 Proteases

The action of antiviral and antibacterial peptides can be hindered by the presence of proteolytic enzymes (proteases) or other proteins in the biological milieu. for example, LL37 is degraded by Chlamydia secreted protease, which is known protease chlamydial protease-like activity factor (CPAF) [154]. Therefore, the use of protease inhibitors can increase its anti-chlamydial efficiency. However, proteases like phosphatase 67 cleave peptides, leading to the separation of the blocking fragment and subsequently the activation of the peptide [154].

### 5.6 Microbial AMP resistance mechanisms

Pathogens represents various resistance mechanisms toward AMPs, complicating treatment options. Enzymatic degradation, membrane charge or fluidity alternation, extrusion by efflux pump, and biofilm formation are common resistance strategies [155]. Monitoring these resistance mechanisms is crucial for the ongoing development of peptide-based therapies.

## 6 Conclusion

Concerns regarding drug resistance and the limited availability of effective treatments for certain viral and bacterial infections have intensified the focus on developing alternative therapeutic options. Cationic peptides, derived from natural sources, have been extensively studied by researchers and pharmaceutical manufacturers as promising antimicrobial agents. This review has provided a comprehensive overview of the inhibitory roles of antimicrobial viral peptides (AVPs) (pre-cell entry and post-cell entry) and antimicrobial bacterial peptides (ABPs) (extracellular and intracellular).

The insights gained from this review are invaluable for the design and synthesis of target-specific peptides, addressing the side effects associated with currently approved peptides, and expanding their application in viral-bacterial co-infections. These data facilitate the development of target-specific peptides, addressing the side effects of existing peptides, expanding their use in viral-bacterial co-infections, enhance their roles as immune modulators, development of synergistic combined therapies, determination of resistance mechanisms, and enable structural modifications to alter peptide action. Future studies should leverage advanced techniques, such as artificial intelligence, to predict mechanisms of action, elucidate structure-function relationships, and understand resistance mechanisms related to peptide activity. Investigating in vivo activity of these peptides, particularly those with dual antibacterial and antiviral properties in co-infections (e.g., HIV-Mycobacterium tuberculosis), as well as their potential in combination with existing drugs or compounds like nanoparticles, will significantly advance the development of new peptide-based therapeutics.

Additionally, research should also consider broadening the scope to include antifungal, antiparasitic, and possibly anti-cancer applications, enhancing the clinical reach of these biomolecules.

## CRediT authorship contribution statement

**Mahdyeh Neghabi Hajigha:** Writing – review & editing, Writing – original draft, Visualization, Data curation. **Bahareh Hajikhani:** Writing – review & editing, Validation, Investigation. **Maryam Vaezjalali:** Writing – review & editing, Validation. **Hossein Samadi Kafil:** Writing – review & editing, Visualization, Validation. **Raana Kazemzadeh Anari:** Writing – review & editing. **Mehdi Goudarzi:** Supervision, Conceptualization.

## Data availability statement

No data was used for the research described in the article.

## Funding

This work is financially supported by a research grant from the 10.13039/501100005851Research Deputy of Shahid Beheshti University of Medical Sciences, Tehran, Iran [Grant No. 3008825]**.**

## Declaration of competing interest

The authors declare that they have no known competing financial interests or personal relationships that could have appeared to influence the work reported in this paper.
